# Ovarian Cancer and Cancer Stem Cells—Cellular and Molecular Characteristics, Signaling Pathways, and Usefulness as a Diagnostic Tool in Medicine and Oncology

**DOI:** 10.3390/cancers13164178

**Published:** 2021-08-19

**Authors:** Andrzej Nowicki, Magdalena Kulus, Maria Wieczorkiewicz, Wojciech Pieńkowski, Katarzyna Stefańska, Paulina Skupin-Mrugalska, Rut Bryl, Paul Mozdziak, Bartosz Kempisty, Hanna Piotrowska-Kempisty

**Affiliations:** 1 Department of Toxicology, Poznan University of Medical Sciences, 60-631 Poznan, Poland; andrzej.m.nowicki@gmail.com; 2 Department of Veterinary Surgery, Institute of Veterinary Medicine, Nicolaus Copernicus University in Torun, 87-100 Torun, Poland; magdalena.kulus@umk.pl (M.K.); bkempisty@ump.edu.pl (B.K.); 3 Department of Basic and Preclinical Sciences, Institute of Veterinary Medicine, Nicolaus Copernicus University in Torun, 87-100 Torun, Poland; maria.wieczorkiewicz@umk.pl; 4 Division of Perinatology and Women’s Diseases, Poznan University of Medical Sciences, 60-535 Poznan, Poland; wpienkowski@poczta.onet.pl; 5 Department of Histology and Embryology, Poznan University of Medical Sciences, 60-781 Poznan, Poland; k.stefanska94@o2.pl; 6 Department of Inorganic and Analytical Chemistry, Poznan University of Medical Sciences, 60-780 Poznan, Poland; psmrugalska@ump.edu.pl; 7 Department of Anatomy, Poznan University of Medical Sciences, 60-781 Poznan, Poland; rutbryl@gmail.com; 8 Department of Poultry Science, North Carolina State University, Raleigh, NC 27695, USA; pemozdzi@ncsu.edu

**Keywords:** ovarian cancer, cancer stem cells, molecular markers, ovarian tumor

## Abstract

**Simple Summary:**

Ovarian cancer is still a high-risk, metastatic disease, often diagnosed at a late stage. Difficulties in its treatment are associated with high resistance to chemotherapy and recurrence. Responsible for the malignant features of cancer are considered to be cancer stem cells (CSCs), which generate new cells by modifying various signaling pathways. Signaling pathways are crucial for the regulation of epithelial-mesenchymal transition, metastasis, and self-renewal of CSCs. New therapies based on the use of inhibitors that block CSC growth and proliferation signals are being investigated. The current histological classification of ovarian tumors, their epidemiology, and the recent knowledge of ovarian CSCs, with particular emphasis on their molecular basis, are important considerations.

**Abstract:**

Despite the increasing development of medicine, ovarian cancer is still a high-risk, metastatic disease that is often diagnosed at a late stage. In addition, difficulties in its treatment are associated with high resistance to chemotherapy and frequent relapse. Cancer stem cells (CSCs), recently attracting significant scientific interest, are considered to be responsible for the malignant features of tumors. CSCs, as the driving force behind tumor development, generate new cells by modifying different signaling pathways. Moreover, investigations on different types of tumors have shown that signaling pathways are key to epithelial-mesenchymal transition (EMT) regulation, metastasis, and self-renewal of CSCs. Based on these established issues, new therapies are being investigated based on the use of inhibitors to block CSC growth and proliferation signals. Many reports indicate that CSC markers play a key role in cancer metastasis, with hopes placed in their targeting to block this process and eliminate relapses. Current histological classification of ovarian tumors, their epidemiology, and the most recent knowledge of ovarian CSCs, with particular emphasis on their molecular background, are important aspects for consideration. Furthermore, the importance of signaling pathways involved in tumor growth, development, and metastasis, is also presented.

## 1. Introduction

Globally, ovarian cancer incidence and mortality rates have not significantly changed over the last three decades. Ovarian cancer is the seventh most frequently diagnosed cancer type in the world, and second, after breast cancer, in women from highly developed countries [1,2]. Furthermore, ovarian cancers are the fifth leading cause of death in women and the most lethal of gynecologic oncology [2]. Due to the fact that afflicted women remain asymptomatic, they are often diagnosed at an advanced stage, which results in a five-year survival rate below 45% [2,3]. The ovary is an organ with a significant number of cells of different origins. Advanced processes concerning both folliculogenesis and oogenesis, as well as the production of sex hormones, require cooperation of many cell types. Neoplasms are formed from almost every part of the ovary. The source of adenomas and adenocarcinomas are epithelial cells. Depending on the subtype of the tumor, the epithelium of the ovarian surface or adjacent organs (fallopian tube, uterus) may undergo neoplastic transformation. Teratomas, dysgerminomas, yolk sack tumors, or choriocarcinomas can originate from the ovarian parenchyma. Furthermore, the most diversified group of neoplasms originate from the ovarian stroma or/and sex cord cells, such as granulosa cell tumors, Sertoli- Leydig cell tumors, fibromas, and thecomas.

As most significant genetic risk factors, previous family history of the disease (3–7-fold increase), mutations within the *BRCA1* and *BRCA2* genes, and Lynch’s syndrome are considered [1,3,4,5]. Predisposing factors for ovarian cancer also include non-Hispanic ethnic group [3] patients’ age over 40 years (with the exception of germ cell tumors, which are more frequently diagnosed in young women [3,6]). The risk of ovarian cancer is linked to continued ovulation, so women who begin ovulating at a young age, do not have children, and reach menopause after age 50 are more susceptible [7]. Furthermore, non-genetic predispositions include obesity, diabetes, smoking, or drugs, as well as certain medicines, including hormone replacement therapy (HRT) [1,2,3,4,5,6,8].

The most recent findings indicate that there is a population of cells in cancer tissue with the capacity for self-renewal and malignant potential. This group is known as cancer stem cells (CSCs), as it was demonstrated that they show the presence of markers typical for stem cells (SCs). It is possible that CSCs are responsible for the activation of the tumor growth, as well as support its expansion [9]. CSCs are described as populations able to renew, proliferate, and maintain cancer even after treatment. Some authors also define these cells as tumor-initiating cells (TICs) [10]. CSCs, as the driving force behind tumor development, generate new cells through the modification of different signaling pathways [11]. External environmental factors can affect stem cells, which are transformed by oncogenic mutations. The formation of metastases is a very complex process involving epithelial–mesenchymal transition (EMT) [12]. This allows the cancer cells to enter the blood vessels, first causing local metastases, the acquisition of migrating properties, and the colonization of distant tissues.

Among gynecological oncological patients, the highest mortality rate concerns ovarian cancer (OC) [13]. In view of the still high mortality rate for ovarian cancer, it is required to develop new diagnostic tools, treatment methods, and successful therapy. Studies on the molecular characteristics of CSCs and their signaling pathways lead to the hypothesis that they are closely associated with disease relapse and treatment resistance. It seems obvious, therefore, that a focused therapy aimed specifically at ovarian CSCs could become a milestone in oncological medicine. However, before a new treatment can be introduced, specific and precise molecular-based diagnostics should be established.

## 2. Histological and Molecular Characteristics of the Ovarian Cancer

### 2.1. Ovarian Epithelial Tumors

A mature ovary is covered by a monolayer of mesothelial cubic epithelium. In contrast, the rest of the female reproductive system, for example, the fallopian tubes, the endometrium, and the vagina originate from the Müllerian ducts, hence are covered by Müllerian epithelium [14]. Initially, the occurrence of different histological types, such as serous, mucinous, clear cell, or endometrioid carcinoma, was previously explained as the result of metaplastic differentiation of the ovarian surface epithelium (OSE) cells. Thus, tumors of different types were eventually supposed to resemble histological tissues of the fallopian tube, endometrium, or cervical canal [15]. However, it is now accepted that these tumor types are distinct entities with different origins, clinical, and biological behavior. The analyses of gene expression profiles confirm the relatedness of particular histological types of ovarian cancer to the normal epithelium of various tissues. It is concluded that the expression pattern of specific genes that characterize correlations between serous carcinoma and fallopian tube epithelium, endometrioid and clear cell carcinoma and uterine epithelium, and mucinous carcinoma and colorectal epithelium as primary rather than secondary [16], shedding new light on the origin of specific types of EOTs.

Epithelial ovarian tumors (EOTs) are classified into histological subtypes, according to the predominant pattern of differentiation of the tumor cells. These subtypes are: serous (70–80%), endometrioid (10%), clear cell (10%), mucinous (3%), and others such as transitional cell tumors (Brenner tumors) and mixed epithelial tumors [1,2]. Neoplasms within each of these groups, depending on the degree of cells differentiation and some specific molecular signatures, may be classified as malignant, borderline, or benign [17]. The overwhelming majority of EOTs belongs to the group with the highest malignancy potential [18].

Due to the specificity of molecular changes within epithelial carcinomas and the fact that some of them may derive from specific precursors, a division of epithelial ovarian carcinomas into two types was proposed [19]. Type I cancers included: (1) low-grade serous ovarian carcinoma (LG-SOC), (2) endometrioid, (3) clear cell, and (4) mucinous. These tumor types develop from well-recognized precursor lesions such as serous borderline tumors or endometriosis. They are usually limited to the ovary, characterized by slow growth and little tendency to metastasis. In most cases, apart from clear cell carcinoma, the prognosis is promising for the patient. In type I tumors, their somatic mutations can be observed in the *KRAS, BRAF, PTEN, PIK3CA, CTNNB1, ARID1A*, and *PPP2R1A*, and very rarely in the *TP53* gene (Table 1) [19]. Type II tumors include high-grade serous ovarian carcinoma (HG-SOC), which are characterized by very rapid growth and high aggressiveness, with the diagnosis very often made at an advanced stage and associated with poor prognosis. In approximately 95% of such cases, mutations in the *TP53* gene are observed. In type I tumors, the mutations are described as rare. In about half of HG-SOC cases, inactivation of the *BRCA1/2* genes is also observed (Table 1) [19].

For many years, CA125 has been the main biomarker for the risk assessment and therapy of women with EOTs [29,30]. Jackson et al. studied the expression of *CA125* in laying hens, which by continually ovulating and laying eggs, often developed ovarian cancer [31]. They found that ovarian cancer cells from laying hens expressed *CA125*, suggesting the possibility of using hens as a model to study female ovarian cancer. However, some novel markers have recently become available to improve the sensitivity and specificity of the diagnosis. The most recent studies, using integrated bioinformatics analysis, identified some differentially expressed genes (DEGs) associated with the EOTs [32,33]. Zhang et al. found three hub genes: *BUB1B, KIF11*, and *KIF20A*, whose upregulation may tend to the lower survival rates and be relevant to prognosis. *BUB1B* expression level was correlated with ovarian FIGO (International Federation of Gynecology and Obstetrics) stage and tumor differentiation. For *KIF20A*, the expression level was correlated with the FIGO stage and intraperitoneal metastatic potential [32]. Some authors also assign a key role in tumorigenesis and prognosis assessment for patients with EOTs to the deregulation of *KIF4A, CDC20, CCNB2, TOP2A, RRM2, TYMS, BIRC5, FOXM1, PSAT1, TRIP13, RAD51AP1, DLGAP5, FAM83D, PRC1, CDCA5, CKS2, MELK, KIF15, CEP55, TTK, UBE2C, CENPF,* and *KIF14* [34,35,36].

### 2.2. Ovarian Germ Cells Tumours

Ovarian germ cells tumors (OGCTs), deriving from the primitive germ cells of the embryonic gonads, are a histologically heterogeneous, mostly benign subtype [37]. Furthermore, the group of germ cells tumors with malignancy potential includes some immature teratomas, dysgerminomas, yolk sac tumors, choriocarcinomas, and mixed germ cell tumors [37].

### 2.3. Stromal and Sex Cord Cells Tumours

Sex cord and stromal cells tumors (SCSTs) are a heterogeneous group and consist of diverse cells arising from the gonadal primitive sex cords cells (granulosa cells and Sertoli cells) or stromal cells (theca cells, fibroblasts, and Leydig cells). These cell types may occur separately (pure sex cord cells or pure stromal tumors) or together and may be characterized by various degrees of differentiation [38,39]. In most cases, depending on the origin of the cells, specific clinical symptoms associated with increased hormone production are observed. Tumors formed from granulosa or theca cells usually induce hyperestrogenicity, whereas those of testicular origin (Sertoli or Leydig) may be hyperandrogenic. In rare cases, these phenomena are reversed or do not occur [38].

## 3. Cancer Stem Cells and Characteristic of Ovarian Cancer Stem Cells

### 3.1. Cancer Stem Cells

Cancer stem cells (CSCs) are a population of undifferentiated cells with unique abilities of self-renewal, proliferation, differentiation, and multipotency. Recent research indicates the fundamental role of the CSCs in the process of carcinogenesis, chemo-resistance, and the formation of metastases [40]. A currently accepted theory states that CSCs arise as a result of ordinary stem cells’ genetic mutations caused by genetic instability and/or adverse effects of the microenvironment [41,42,43]. Additionally, acquired mutations of the CSCs, together with the capacity for self-renewal, can lead to the progression of malignancy [40,42,44]. CSCs have been identified for the first time in acute myeloid leukaemia (AML) by Bonnet and Dick in 1997, who showed that a single leukemic cell was able to transmit systemic disease when transplanted into the severe combined immune-deficient (SCID) mice as recipients [45]. Their presence was subsequently found in many types of solid tumors in the breast, brain, head and neck, liver, lungs, pancreas, prostate, or ovaries [46].

Each cell, including stem cells, can be identified by the expression of a specific molecule or combination of molecules called markers [40,41,47]. Proper SCs and CSCs have a common range of multiple molecules on the surface of cells belonging to the cluster of differentiation group (CD) such as CXCR4, Sca-1, CD133, CD24, CD34, CD44, c-kit, c-met, LIF-R, and BMI1. Importantly, defining CSCs solely on the basis of the appearance of surface markers is insufficient, as none of the markers is found exclusively on CSCs [40]. Presently, there is no universal marker that identifies the CSC of each tumor [40,42]. Studies on glioma stem cells, which show both CD133+ and CD133− phenotypes, depending on the part of the tumor analyzed [48], confirm that there is no universal CSC marker. Similar genetic divergence has been shown for breast cancer stem cells of CD24−/CD44+ and CD24+/CD44− phenotype [49]. These data may indicate both the known fact of genetic heterogeneity of the tumors and the existence of continuous selection of cells best adapted to a given environment [42,48,49]. The concentration of cytoprotective enzymes, e.g., aldehyde dehydrogenase (ALDH) or the expression level of ATP-binding cassette (ABC) transporters, are also analyzed [40,41].

**Figure 1:**
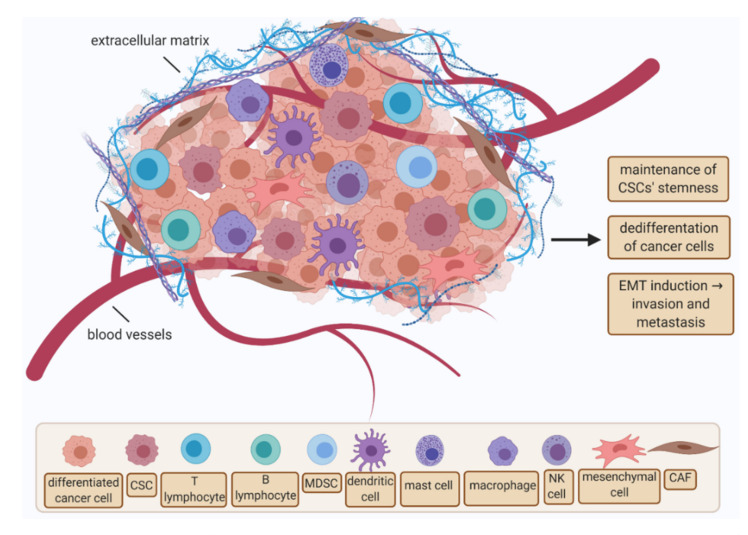
Cancer stem cells’ microenvironment—niche. Abbreviations: CAF—cancer-associated fibroblast; EMT—epithelial-to-mesenchymal transition; CSC—cancer stem cell; MDSC—myeloid-derived suppressor cell; NK cell—natural killer cell (created with BioRender.com).

One of the factors directly influencing the maintenance of the stem cell population is the niche microenvironment (Figure 1) [50,51]. The niche is formed by various stromal cells, vascular networks, mesenchymal cells, immune system cells, extracellular matrixes (ECM), and factors secreted by these cells [50,52]. The microenvironment can maintain CSCs not only in the state of the stemness but also directly affect the differentiation of normal cells into the CSCs and induce the epithelial to mesenchymal transition (EMT), which results in the high potential of invasion of the cancer cells and their ability to the metastases formation [50]. It has been indicated that it is the epigenetic changes associated with EMT that affect the phenotypic differences between the CSCs and the other cells forming the tumor mass. This phenotypic diversity of cancer cells determines the development of resistance to different types of therapy. In addition, EMT mechanisms are most likely associated with relapse [53,54]. Both genetic and molecular mechanisms and factors of the specific microenvironment of the neoplastic niche may contribute to the formation of phenotypically different cells in the tumor mass. To maintain their properties and ability to regenerate, stem cells need signals from the cells of the niche in which they occur. Many studies indicate that the control of self-renewal and proliferation and/or drug resistance of CSCs is the responsibility of certain factors and intracellular metabolic pathways [40,50].

The EMT is based on epigenetic changes that are phenotypically revealed, hereditary, and unrelated to genetic alterations. Morphological changes of cancer cells are observed, such as shape transformation from epithelial (cuboidal, columnar) to elongated, fibroblast-like, with subsequent loss of cell-cell connections and apical-basal polarity. These acquired features allow for greater cell mobility, which affects their invasiveness [55,56,57]. Physiological, cytoskeletal composition, and ECM changes are also observed. While cell-cell junctions in epithelial cells (ECs) are clearly marked, tight, adherent, gap junctions and desmosomes predominate between cancer cells. Mesenchymal cells (MCs) have a negligible number of connections. The characteristic adhesion belt in ECs is produced by actin fibers that attach to adherens junctions. In MCs, the cytoskeleton is mainly represented by actin stress fibers and vimentin intermediate filaments. The changes also affect the ECM, where the ECs are placed on a basement membrane made of laminin and collagen IV [58]. Furthermore, interactions take place through integrin α_6_β_4_ on hemidesmosomes. During EMT, the cells establish a reaction via β_1_ or β_3_ containing integrins in adhesion plaques. ECM in MCs is mainly composed of collagen I and fibronectin [59,60].

### 3.2. Ovarian Cancer Stem Cells

A thorough analysis and understanding of the molecular basis of the ovarian tumor microenvironment, especially studies of its transcriptome and proteome, may provide a basis for therapeutic strategies for ovarian tumors. Moreover, ovarian cancer stem cells and their reactions with a specific niche are not without significance in this context. In principle, the elemental richness of the ovarian cancer microenvironment influences different pathways and direction of lesion progression [61]. The interaction of cells, through the secretion of different molecules and signals, models the niche and influences tumor development. Furthermore, the specific extracellular matrix (ECM) is an important component of the tumor microenvironment, as it is composed of, e.g., inflammatory cytokines, integrins, chemokines, and matrix metalloproteinases (MMPs).

Stromal cells make up a significant portion of the tumor, including cancer cells, cancer stem cells, cancer-associated fibroblasts (CAFs), pericytes, immune cells, and endothelial cells (ECs). CAFs are cells of mesenchymal origin, although they can also undergo transdifferentiation from other lineages, such as ECs or epithelial cells. Exposure to factors such as TGF-β (tumor-derived transforming growth factor-β,) bFGF (basic fibroblast growth factor), PDGF (platelet-derived growth factor), MMPs, reactive oxygen species, and VEGF (vascular endothelial growth factor) is essential in this process [62,63,64]. CAFs are the source of most protein components of ECM (collagen, laminin, fibronectin), as well as secrete TIMPs and MMPs [64]. Furthermore, various mechanisms through which CAFs promote tumor progression have been described [65,66,67]. Tumor-derived fibroblasts have long been recognized as cells that increase the malignancy of tumors, while transplanted tumor cells, together with CAFs, resulted in faster changes than those caused by cancer cells alone [68,69]. Stromal derived cells are also known to affect the metabolism of CSCs and induce consequences related to malignancy and the possibility of metastasis [70]. A recent study showed that OCSCs cooperate with macrophages of the tumor niche to promote tumor malignancy [71]. First, the upregulation of M2 macrophage marker CD206 led to activation of immunosuppressive programs, as well as increased ALDH activity, which consequently activated pro-tumoral activity and self-renewal of OCSCs. Increased levels of pro-inflammatory cytokines (IL-10 and IL-6) were also observed. It was assessed that macrophage-initiated WNT signaling might contribute to a more aggressive ovarian cancer phenotype, in this case, prompting the use of this pathway in targeted therapy [71].

However, the metabolic processes associated with OCSCs are not yet fully understood. The predominant metabolic mechanisms depend on the relationships between individual elements of the tumor microenvironment, as well as current energy requirements. The plasticity of OCSCs is also an important aspect in a range of metabolic processes [72]. A recent study showed that OCSCs that were detached from the in vitro surface and suspended in ascites changed their metabolism from glycolysis to increased lipid metabolism. In addition, the expression of the CSCs markers, such as cluster of differentiation 44 and c-kit, was also demonstrated [73]. Therefore, a thorough understanding of the characteristics of OCSCs metabolic processes seems to be one of the key elements in assessing their potential for malignancy and targeted therapy. CSCs contribute to the difficulty of treatment of ovarian cancer, as resistant cells tend to persist after chemotherapy, potentially causing tumor recurrence. They exhibit altered lipid metabolism, resulting in lactate accumulation and acidification, which causes T cell dysfunction [74]. Moreover, it has been suggested that CSCs may be formed on the background of metabolic changes occurring in non-CSCs cells [75].

## 4. Ovarian Cancer Stem Cells as a Useful Diagnostic Tool—A Role in Metastasis

**Figure 2:**
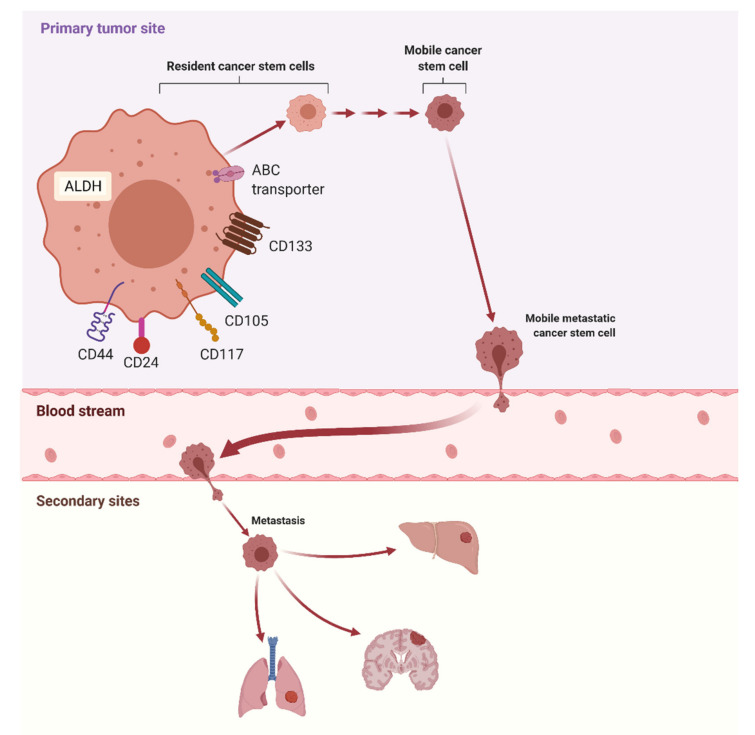
Potential markers of cancer stem cells (CSCs) and the role of CSCs in metastasis. Abbreviations: ALDH—aldehyde dehydrogenase (created with BioRender.com).

It is claimed that CSCs are responsible for the spread of neoplasms and the formation of metastases to the abdominal cavity. Unlimited growth and providing a source for most of the different cells that build the tumor mass are the characteristics of CSCs, which usually form a small part of the tumor [76]. The results of various studies indicate that there are CSCs markers that are supposed to play a key role in the formation of metastases (Figure 2). It is also suggested that signaling pathways, such as WNT, Notch, and Hedgehog (Hh), are important in the EMT process. Hence, genetic evaluation creates many diagnostic possibilities. It is possible to use CSCs markers to classify the tumor and choose the therapeutic treatment. Identification and isolation of CSCs from tumors are possible, e.g., through magnetic-activated cell storing method (MACS) and fluorescent-activated cell storing (FACS), based on both surface and intracellular markers. Monoparameter isolation (MACS) is the faster of these methods, while multi-parameter separation (FACS) gives more possibilities. Hence, as specific markers for other cancers, including blood cancers, have already been identified, it seems crucial to identify those for ovarian CSCs.

Ovarian cancer stem cells (OCSCs) are characterized by the presence of CD44, CD117, CD133, and CD24, as well as ALDH activity [77,78,79,80]. Mutations taking place in genes encoding proteins entering the major pathways that are known to be active in OCSCs, i.e., Hedgehog, Wnt, Notch, etc., may have a significant impact on the formation of ovarian cancer and metastases [77,80].

### 4.1. CD133

Among cellular surface markers, CD133 is the most commonly used to isolate CSCs [13]. In addition, it has been indicated that the presence of CD133 in cancer tissue may be associated with increased malignancy of the tumor [81]. Accelerated growth of renal tumor was observed after transplantation of CD133+ cells, which were involved in the neovascularization processes [82]. However, some studies have shown that both CD133+ and CD133− cells from lung cancer (A549, H446) exhibit features of self-regeneration and malignancy, as well as resistance to chemotherapy [9]. Furthermore, similar conclusions have been described for the glioma cancer stem cells (C6) [83]. Hence, a conclusion was made that the presence or absence of CD133 alone cannot indicate a classification of the tumor as malignant. Following implantation of the CD133+ cell fraction, the ovarian tumors obtained consisted of both CD133+ and CD133− cells. However, CD133+ cells showed a higher tumorigenic capacity than CD133− cells. This leads to the conclusion that these positive cells may differentiate into various phenotypic populations [84]. Additionally, CD133− cells were shown to also generate tumors that consisted of both cell populations (positive and negative). However, it is not clear whether if the CD133− cells that have the ability to give rise to CD133+, or if their occurrence is caused by the presence of a small fraction of CD133+ cells among the purified CD133− fraction. Another possibility is to produce the CD133+ cells from a different population of tumor CSCs [78]. Furthermore, the presence of CD133 marker on cells originating from ovarian cancer has been analyzed by many research teams [85,86,87]. CD133+ cells were identified in both benign and malignant tumor cells but also in normal ovarian tissue. However, the percentage of these cells in primary ovarian tumors was usually higher than in normal tissue or metastases [85]. The use of tissue microarrays proved that the expression of CD133 was associated with a high grade of serous ovarian carcinoma, ascites, late stages of the disease, and no response to treatment [87,88].

### 4.2. CD105

A well-known marker of mesenchymal stem cells (MSCs) is CD105, which plays a role in the angiogenesis processes, working as a surface cell receptor for transforming growth factor (TGF). CD105+ cells isolated from human renal tumor tissues and implanted in mice showed tumorigenic properties [89]. In addition, it was also found that the CD105+ cancer cells showed increased expression of other MSCs markers (CD73 and CD90), while decreased expression was marked for CD44 and CD146. At the same time, it is suggested that the CD105 cell marker is a temporary and transient factor since only half of the CD105+ tumor cells were shown to exhibit its expression after in vitro culture [10]. A study by Zhang et al. [90] revealed that high expression of CD105 was associated with drug resistance, advanced disease stage poor differentiation and high rate of recurrence in epithelial ovarian cancer.

### 4.3. CD44

CD44 is considered a controversial surface antigen of CSCs. It plays a role in many different physiological processes, including growth, cell differentiation, and wound healing, but it has also been shown to be involved in neoplastic metastases [91]. Biochemically, CD44 is a transmembrane glycoprotein that binds extracellular glycosaminoglycan hyaluronate [92]. Some data indicate a correlation between CD44 expression and metastases [93], as well as resistance to chemotherapy [94]. Furthermore, the subpopulation of CD44+ cells has been shown to retain the ability to initiate ovarian tumor after immortalization [44,95]. Additionally, a population of CD44+ CD24− ovarian cancer cells was observed and characterized by resistance to chemotherapy. These cells had the ability to form spheroids and presented a higher risk of relapse [96,97]. It is also suggested that the CD44+ CD117+ cells have a higher tumorigenicity than the CD44− CD117− cells [98]. Subsequent studies also indicate that only CD117+ ovarian tumor cells have the ability to initiate the tumor [99]. The CD24 superficial cell marker was used to identify cancer cells of different tissues. Gao et al. described that CD24+ cells showed greater resistance and ability to self-regenerate than CD24− cells [100].

### 4.4. ALDH

The activity of aldehyde dehydrogenase (ALDH), determined using Aldefluor method, was identified as yet another important marker of CSCs. The ALDH enzyme plays a role in detoxification by oxidizing aldehydes and converting retinol to retinoic acid. Double positive CD133+ and ALDH+ cells in ovarian tumors [101] showed resistance to chemotherapy and increased growth and initiation of tumors in mice [102]. The ALDH1 isoform was also tested for its usefulness as a marker of CSCs for ovarian cancer. However, the results were of these studies was inconsistent [103,104]. The association of ALDH1 (br) expression with CD44 in ovarian cancer cells was shown to increase resistance to treatment [105], whereas the results obtained by Chang et al. indicate that ALDH1 was a positive prognostic factor in ovarian cancer therapy [106].

It is possible to isolate CSCs based on the side population (SP) with the expression of the ABC transporter [78]. In addition, specific CD44+/MyD88+ epithelial ovarian cancer stem cells have been shown to be responsible for tumor initiation, even after surgical and chemotherapeutic treatment. The repair and self-renewal of this tumor are promoted through the pro-inflammatory TLR2-MyD88-NFκB pathway [107]. Another study suggests an important role for HMGA1, a chromatin remodeling factor, in ovarian cancer stem cell function [108]. The study was based on a 3D culture of A2780, SKOV3, and PA1 ovarian cancer cells, where elevated HMGA1 expression was observed along with expression of stemness markers. The HMGA1 knockout decreased proliferation, spheroid formation ability, and stemness marker expression, as well as resistance to chemotherapy [108].

Using the FACS method, it is possible to sort OCSCs by specific markers. The isolated cells showed typical phenotypic features for CSCs (including therapy resistance, self-renewal, and tumor growth). However, no tumor development was observed from sorted CSCs that mimicked the progression typical of human ovarian cancers [109,110], although this could occur due to a number of internal and external factors, as well as the way the isolated cells were transplanted subcutaneously into mice. In human patients, the most common metastases involve the nearest environment to the ovary (including the peritoneum, mesentery, bladder, colon, and others). In addition, differently stimulated human organisms and the reduced immune response of experimental mice are not insignificant [111]. The tumorigenic potential of OCSCs depends on the distinct profile of specific markers. However, differences in genetic, epigenetic, or molecular background between populations of CSCs that have distinct marker profiles remain to be established. In addition, whether it is possible to reproduce the original tumor heterogeneity after transplantation of isolated OCSCs into a mouse model has not been accurately determined [112]. The potential of OCSCs to metastasize and form spheroids may account for the spheroid complexes in the peritoneal cavity of patients with advanced disease [113].

The complexity of ovarian cancer and the confirmed presence of typical CSC markers classified indicate that identification of a single marker does not confirm OCSCs presence. However, knowing the entire marker profiles and biochemical properties may determine the populations of these cells. So far, it has not been possible to determine a CSC marker that would only occur in the ovary, as the markers discovered to date are specific to other various cancers. The studies mentioned above show that the presence of CSCs markers on ovarian cancer cells was usually associated with higher malignancy and risk of relapse and resistance to treatment, which was consequently associated with a poorer prognosis for the patient. This leads to continued research in this direction, the search for specific markers for ovarian CSCs, and the analysis of the involved signal pathways, which may provide new diagnostic and therapeutic tools.

### 4.5. Treatment Approaches against CSCs

Research into the effectiveness of treatment methods for ovarian cancer is key to reducing disease recurrence and achieving complete remission. Two main treatment approaches to overcome CSCs have been proposed. The first approach aims at the loss of their ability to self-replicate (i.e., induction of their differentiation), e.g., through the use of retinoic acid. The second option is targeted therapy, based on inhibitors of signaling pathways (WNT, Notch, SHH) [114].

Cisplatin, targeting CD44+/CD117+ cells overexpressing CXCR4, was used to inhibit metastasis and limit invasion of cancer-initiating cells [115]. However, a study by Abubaker and co-authors showed that a single treatment with cisplatin and paclitaxel-based chemotherapy omits residual cells with CSCs characteristics, which increases the metastatic potential [116]. Additionally, another work [117], based on mouse xenograft models injected with PKH26-labeled SKOV3 ovarian cancer cells, also supports the above conclusions. While it was shown that cisplatin caused inhibition of tumor growth, numerous dormant cell clones appeared. These cells, arrested at the G0/G1 stage, also showed high levels of stemness markers (Oct-4, Nestin, CD117, CD44). Chemotherapy with cisplatin led to the enrichment and strengthening of stemness features in epithelial ovarian cancer cells [117]. One possibility for reducing CSCs resistance to cisplatin is the use of salinomycin, which induces apoptosis in ovarian cancer cells [118]. The apoptosis-inducing activity of salinomycin was also demonstrated on an ovarian cancer cell line (OVCAR-3) [119]. OCSCs isolated from the OVCAR-3 ovarian cancer cell line, expressing CD44+ and CD117+ markers that have been associated with chemo-resistance, inhibited growth after treatment with salinomycin and paclitaxel [120]. The action of salinomycin to initiate apoptosis in ovarian cancer stem cells may be related to death receptor 5 (DR5) and caspase 8 [121], as well as nuclear transcription factor NF-κB [122]. Furthermore, interesting effects have been observed in diabetic women diagnosed with ovarian cancer. Because of their diabetes, they were taking metformin, which inhibits the growth of CSCs, thus improving cancer outcomes compared to women not taking metformin. Shank et al. [123] showed, using the FACS method, that metformin reduces ALDH+ OCSCs and tumor sphere formation. In addition, similar results were obtained in studies on ovarian cancer cell lines SKOV3 and A2780 [124]. Low doses of metformin reduced the CD44+, CD117+ population, as well as affected EMT inhibition, which in result enhances the effect of chemotherapy. Clostridium perfringens enterotoxin (CPE) may be an alternative option to eliminate ovarian cancer stem cells. CD44+ OCSCs highly expressing claudin 4 underwent apoptosis after exposure to CPE [125,126]. There are also reports that the phenotype of ovarian cancer-initiating cells may be related to PKCι, which may be a target of auranofin in cancer therapy [127].

## 5. Signaling Pathways Involved in Ovarian Carcinogenesis

**Figure 3:**
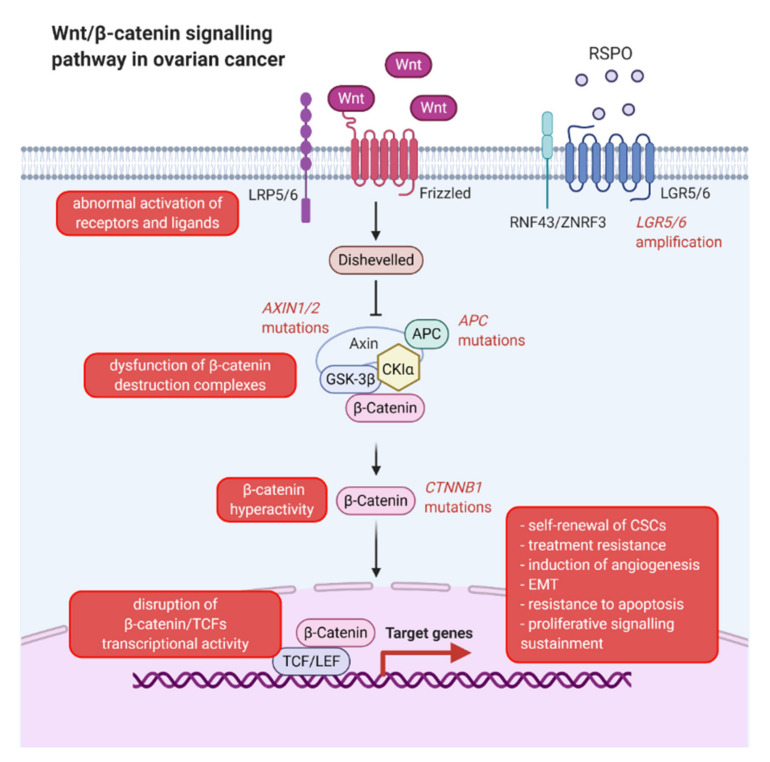
Wnt/β-catenin signaling pathway in ovarian cancer. Abbreviations: APC—adenomatosis polyposis coli; CKIα—casein kinase 1α; CSC—cancer stem cell; EMT—epithelial-to-mesenchymal transition; GSK-3β—glycogen synthase kinase 3β; LGR5/6—leucine-rich repeat-containing G-protein coupled receptors 5/6; LRP5/6—low-density-lipoprotein-related proteins 5/6; RNF43/ZNRF3—RING finger protein 43/Zinc/Ring finger protein 3; RSPO—R-spondin; TCF/LEF-T—cell factor/lymphoid enhancer-binding factor proteins; Wnt—An acronym standing for homologous wingless (wg) and Int-1 (created with BioRender.com).

Throughout research on effective methods of treatment of chemotherapy-resistant ovarian cancers, signaling pathways implicated in the course of carcinogenesis should be analyzed, as their modification may be crucial to inhibit CSCs activity [128]. The most notable pathway is the WNT signal transduction pathway (Figure 3), which is highly involved in the process of normal embryogenesis. The research of recent years indicates a correlation between reduced regulation of WNT signaling and the occurrence of diseases [129]. Signals directed extracellularly through the WNT pathway stimulate several intracellular signal transduction cascades. Its ligands, secreted lipid-modified glycoproteins that are rich in cysteine amino acids, are referred to as Wnts [130]. Furthermore, two sub-pathways are distinguished: canonical or WNT/β-catenin dependent (mediated through a transcriptional regulator- β-catenin), and a non-canonical or β-catenin independent [131]. Cellular processes such as polarization, motility, organogenesis, and stem cell regeneration are regulated by WNT proteins. Therefore, their expression is crucial for the proper course of the mentioned processes. Hence, malfunctions in WNT signaling result in various pathologies, often including tumors and birth defects [132]. WNT ligands are bound to the N-terminal domain of the cysteine-rich Frizzled (Fz) receptor family. Additionally, low-density-lipoprotein-related protein5/6 (LRP5/6) is obligatory for this reaction to occur. The signal then moves to cytoplasmic phosphoprotein-disheveled (Dsh/Dvl). This compound causes the ability of the GSK-3β/Axin/APC complex to be inhibited, resulting in the inhibition of the degradation of cytoplasmic β-catenin. This results in its accumulation and transfer to the nucleus, where it binds TCF transcription factors, replacing the Groucho protein, resulting in activation of gene transcription [133]. It has been shown that WNT/B-catenin signal pathways perform a very significant role in the molecular processes of CSCs, as well as in the carcinogenesis of all types of ovarian cancer [134]. The regulation of cell proliferation and apoptosis by WNT/B-catenin target genes implicate them in cancer initiation and progression. Furthermore, this pathway is considered to be involved in epithelial-to-mesenchymal transition (EMT). Additionally, changes of WNT pathway proteins in the cell membrane, cytoplasm, and nucleus are involved in the development of ovarian cancer [135]. The LGR receptors that amplify WNT signaling have been described in relation to the development of high grade serous ovarian carcinoma (HG-SOC) derived from the fallopian tube epithelium [136]. Hyperactivity of β-catenin, in the case of ovarian cancer, and mutations of *AXIN, CTNNB1*, and *APC* genes were observed in epithelial ovarian cancer (EOC). Disorders of β-catenin destruction complexes, incorrect promotion of β-catenin/TCF transcriptional activity, or abnormal activation of receptors and ligands were also observed in the case of ovarian cancer. This pathway has been shown to be associated with the self-regeneration of CSCs, which in consequence was related to resistance to treatment, as well as increased neovascularization [133].

Another important signaling pathway is the sonic hedgehog (SHH), which participates in the regulation of basic molecular processes related to development, embryogenesis, and maintenance of homeostasis in adult tissue and stem cell biology. This pathway has also been associated with various cancers and birth defects [137]. A particular connection has been shown between this pathway and neoplasms such as glioma, lung squamous cell carcinoma, and myeloid leukaemia [138,139,140]. The therapies aimed at the SHH pathway target CSCs, inhibiting their development, differentiation, and proliferation [141]. In the course of the study of SHH pathway abnormalities in cells derived from human ovarian tumors, it was found that the overexpression of Patched and Gli1 was correlated with poorer survival of the patients. This was associated with increased proliferation, motility, and invasiveness of cancer cells due to up-regulation of genes such as E-cadherin, vimentin, Bcl-2, caspases but also beta1 integrin, type 1 matrix metalloproteinase membrane (MT1-MMP), and vascular endothelial growth factor (VEGF) [142]. Similarly, for ovarian teratomas, a strong association with the SHH pathway and Patched and Gli1 proteins was demonstrated [143]. Ke et al. noted that up-regulation of Gli1 promotes epithelial–mesenchymal transition (EMT) in ovarian cancer, increases migration capacity, and causes cross-talk between SHH-Gli1 signals and PI3K-Akt pathway [144]. In addition, a recent study showed a correlation between CD24 expression and SHH regulation, with the possibility of tumor reduction also reported. Hence, the authors suggested that SHH signaling may be a target for inhibition of ovarian cancer progression [145]. Furthermore, high expression of FoxR2 in ovarian cancer stimulates angiogenesis and activates the SHH pathway, which affects the malignant behavior of this cancer. Additionally, up-regulation of FoxR2 was also associated with EMT and cell migration [146]. A recent study indicates that the HH pathway is responsible for resistance to chemotherapy due to its relation with the *MDR1* gene in ovarian cancer [147].

The Notch signaling pathway, one of the main channels of intracellular communication, is implicated in the control of animal cell identity and development processes, including embryogenesis [148]. The ultimate fate of the cells is determined by signals that are exchanged by neighboring cells through Notch receptors, allowing for amplification and consolidation of molecular differences [149]. Notch genes (*NOTCH1, NOTCH2, NOTCH3*, and *NOTCH4*) encode transmembrane receptors for delta-like canonical Notch ligand (DLL1,3,4) signal and jagged canonical Notch ligand (JAG) 1 and 2. Notch signaling activates transcriptions of a number of genes, including BMI1 proto-oncogene polycomb ring finger, cyclin D1, CD44, and MYC. Incorrect activation of this pathway is observed in breast cancer, non-small-cell lung cancer, and hematological malignancies [150].

**Figure 4:**
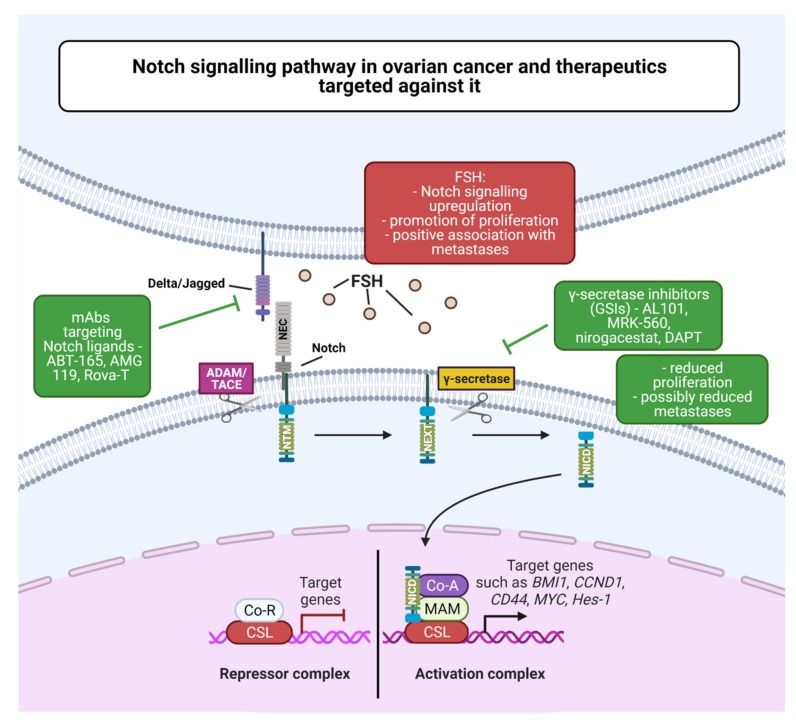
Notch signaling pathway in ovarian cancer and therapeutics targeted against it. Abbreviations: ADAM/TACE—a disintegrin and metalloproteinase/tumor necrosis factor-alpha converting enzyme; BMI1—B lymphoma Mo-MLV insertion region 1 homolog, CCND1—cyclin D1; CD44—CD44 Molecule (Indian Blood Group); Co-A—co-activator; Co-R—co-repressor; CSL—CBF1, Suppressor of Hairless, Lag-1; FSH—follicle-stimulating hormone; Hes-1—hairy and enhancer of split-1; mAbs—monoclonal antibodies; MAM—mastermind; MYC—MYC proto-oncogene, bHLH transcription factor; NEC—Notch extracellular subunit; NEXT—Notch extracellular truncation; NICD—Notch intracellular domain; NTM—Notch transmembrane subunit (created with BioRender.com).

Using genome profiling of serous ovarian carcinoma, it was determined that changes in the Notch pathway are among the most frequently observed [151]. CSCs are known to activate signal transduction paths, including Notch, sparking interest in research into methods of signaling inhibition, which could be crucial for the development of new therapeutic methods (Figure 4). This is particularly important in the context of the Notch pathway contribution to the neoplastic metastasis process. There are two classes of Notch inhibitors: γ-secretase inhibitors (GSIs; AL101, MRK-560, nirogacestat) and monoclonal antibodies (mAbs; ABT-165, AMG 119, rovalpituzumab tesirine (Rova-T)). The first class inhibits Notch receptor cleavage, while the second disrupts ligand–receptor interaction [12]. The study by Akbarzadeh et al. showed that blocking the Notch pathway significantly decreased proliferation of human OVCAR-3 ovarian cancer cells, and treatment with DAPT resulted in a decrease in Hes-1 mRNA concentration and metalloproteinase 2 and 9 activity. The authors conclude that this may reduce OVCAR-3 metastases [152]. Gera and colleagues [149], in their study, showed a relationship of increased expression of Notch and FSH signaling for ovarian cancer and metastases. Spheroids from ascites of affected ovarian cancer patients expressed FSHβ mRNA and secreted this hormone into the medium. In contrast, cells from primary ovarian tumors and cell line monolayers expressed FSHβ at very low levels. The spheroids also showed a higher expression of Notch genes than cell monolayer cultures. The study concluded that spheroids in ascites secrete FSH, which increases the proliferation of cancer cells through Notch signaling, as well as promotes metastasis through autocrine action [153]. A recently published study showed that the interaction of WNT (β-catenin) and Notch signaling promotes proliferation and migration of ovarian cancer cells, mainly involving Jagged 1 [154].

## 6. Therapeutic Approaches Targeting Stem Cell-Associated Pathways

New therapeutic approaches have been introduced to target pathways vital for the maintenance and regulation of stem-like properties. The Hedgehog pathway, which is abnormally initiated in OC, determines cell growth, motility, invasion, and tumorigenesis [155].

Sonidegib is a Hedgehog inhibitor for basal cell carcinoma approved by FDA. The compound was combined with paclitaxel and tested in phase I clinical trial as a therapy for advanced ovarian cancer patients. Sonidegib exhibited anticancer activity, which enabled Stathis et al. to determine a recommended dose for phase II trial [156].

Another Hedgehog inhibitor, Vismodegib, was tested in phase II clinical trial as maintenance treatment for ovarian cancer patients in second or third complete remission (NCT00739661) [157]. Nevertheless, no crucial survival upgrade was noted (5.8 months for placebo vs. 7.5 months for the treatment group), which suggests that Hedgehog pathway arrest is not sufficient to prevent relapse of ovarian cancer [157].

It is well-known that Focal adhesion kinase (FAK) is a protein profusely expressed in CSC. FAK contributes to the interaction with stromal cells to induce intracellular signaling cascades [158]. Defactinib, an inhibitor of FAK, combined with paclitaxel, was shown to have limited activity in ovarian cancer phase I clinical trial. Hence, the CSC niche might be suggested as a proper target for ovarian cancer therapy strategy [159].

Metformin hydrochloride, an anti-diabetic drug used for the treatment of type 2 diabetes, was shown to potentiate chemotherapy effects by targeting CSCs. Metformin was also reported to exhibit a synergy effect with conventional chemotherapeutic agents, decreasing tumor relapse rate [159].

Ipafricept is a well-known inhibitor of the Wnt signaling pathway. Ipafricept, used in combination with carboplatin and paclitaxel, was shown to promote cell differentiation in ovarian cancer xenografts during phase I clinical trial. In total, 82% of relapsing platinum-sensitive ovarian cancer patients responded completely or partially to the treatment [160].

## 7. Conclusions

Ovarian cancers remain the most aggressive gynecologic tumors in women. Despite many productive studies on the understanding of their molecular basis and the identification of various markers, the prognosis for patients is still poor due to frequent recurrences. A small population of cancer stem cells in ovarian cancer seems to be responsible for treatment resistance, poor prognosis, and metastasis. Therefore, an in-depth study of OCSCs is necessary to utilize these cells as a goal in targeted anticancer therapy.

## Figures and Tables

**Table 1:** A molecular characteristic of the main histotypes of epithelial ovarian cancers.

EOC Histotype	Cancer Type	Precursor Lesions	Molecular Changes	Cytogenetic Band	Signaling Pathway	References
HG-SOC	II	serous tubal intraepithelial carcinoma (STIC)	*TP53* mutations *BRCA1/2* mutations amplification of oncogenes *CCNE1*, *NOTCH3*, *RSF1*, *AKT2* *PIK3CA*	17p13.1 17q21/13q12.3 19q12 19p13.2-p13.1 11q14.1 19q13.2 3q26.32	TP53 Notch PI3K/Akt/mTOR PI3K/Akt/mTOR	[19,20]
LG-SOC	I	cystadenoma adenofibroma micropapillary serous carcinoma	*BRAF*/*KRAS* mutations	7q34/12p12.1	MAPK/Erk	[1,17,21]
Endometroid	I	endometriosis endometroid adenofbroma	*PTEN*, *PIK3CA*, *CTNNB1* mutations	10q23.31 3q26.32 3p22.1	PI3K/Akt/mTOR PI3K/Akt/mTOR WNT/β-catenin	[1,17,22] [20]
Clear cell	I	endometriosis clear cell adenofibroma	*ARIDA1A*, *KRAS*, *PPP2R1*, *PIK3CA* and *PTEN* mutations	1p36.11 12p12.1 19q13.41 3q26.32 10q23.31	MAPK/Erk PI3K/Akt/mTOR WNT/β-catenin	[23,24,25,26]
Mucinous	I	mucinous cyst	*KRAS* mutations *HER2*/*neu* amplification	12p12.1 17q12	MAPK/Erk	[17,27,28]
